# TRF2–RAP1 inhibits homology-directed repair of telomeres by promoting BLM-mediated removal of telomere R-loops

**DOI:** 10.1093/nar/gkag272

**Published:** 2026-03-31

**Authors:** Fengshan Liang, Sandy Chang

**Affiliations:** Department of Laboratory Medicine, Yale University School of Medicine, 330 Cedar St., New Haven, CT 06520, United States; Department of Laboratory Medicine, Yale University School of Medicine, 330 Cedar St., New Haven, CT 06520, United States; Department of Pathology, Yale University School of Medicine, 330 Cedar St., New Haven, CT 06520, United States; Department of Molecular Biophysics and Biochemistry, Yale University School of Medicine, 330 Cedar St., New Haven, CT 06520,United States

## Abstract

The telomere long noncoding RNA TERRA forms R-loops *in vitro* at telomeres in a RAD51AP1-dependent manner. In classic DNA double-strand break repair, RAD51AP1 promotes R-loop formation and enables RAD51 to form D-loops by promoting the invasion of local RNA transcripts into donor DNA to form DNA–RNA (DR)-loops. We have previously shown that cells lacking the basic domain of TRF2 and functional RAP1 accumulate telomere D-loops, resulting in homology-directed repair (HDR)-mediated telomere-telomere clustering and formation of ultrabright telomeres (UTs). TRF2^B^ also cooperates with RAP1 to repress telomere R-loop formation in UTs. TERRA has been shown to promote telomere HDR, associating telomeres during R-loop formation. However, the mechanism behind TERRA-mediated telomere HDR and how the TRF2–RAP1 complex regulates telomere R-loops remain unclear. Using reconstituted biochemical systems, we found that RAD51AP1 and TERRA-dependent R-loops promote RAD51-mediated telomere D-loop formation in a TERRA length- and sequence-dependent manner. Specifically, RAD51–ssDNA filaments capture telomere R-loops preferentially over dsDNA. We also discovered that BLM’s interaction with the TRF2–RAP1 complex is required to promote BLM helicase-mediated unwinding of telomere R-loops. Importantly, BLM-deficient cells and cells reconstituted with BLM mutants unable to interact with TRF2 accumulate telomere R-loops in UTs. Our findings highlight a novel mechanism revealing that the TRF2–RAP1–BLM complex removes R-loops at telomeres to inhibit the generation of telomere D-loops, thus repressing telomere HDR and UT formation.

## Graphical Abstract

**Graphical Abstract:**
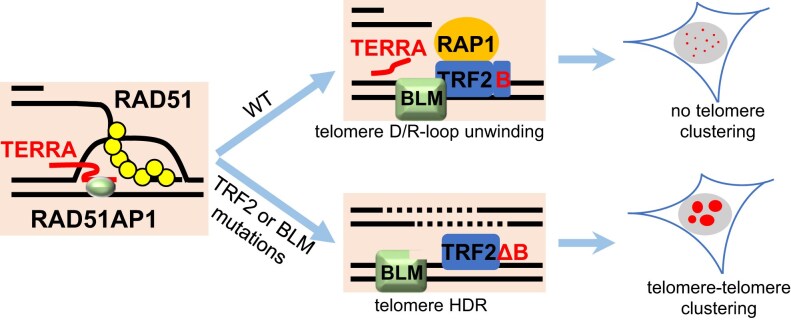

## Introduction

Telomeres are TTAGGG repetitive DNA–protein complexes required to maintain genome stability by safeguarding chromosomal ends from being recognized as DNA double-strand breaks (DSB). Telomeres are protected by six specialized proteins known as the shelterin complex that mediates telomere end protection by repressing the activation of DDR (DNA damage response) pathways [1–5]. The POT1–TPP1 complex binds to the 3′ telomeric single-stranded (ss) G-overhangs and ssDNA–dsDNA junctions to prevent the activation of ATR-dependent DNA damage responses [6, 7]. TRF2 binds to double-stranded telomere DNA and helps fold the 3′-G-rich single-stranded overhang into the dsDNA to form protective telomere T-loops [8, 9] to repress both ATM-dependent DNA damage signaling and telomere repair through the classical nonhomologous end joining (C-NHEJ) pathway [10–12]. TRF2 interacts with RAP1 to prevent telomere ends from activating ATR-dependent homology-directed repair (HDR) [13–15]. During HDR, the RAD51 recombinase forms nucleoprotein filaments on single-stranded (ss) DNA, which in turn captures double-stranded (ds) DNA to search for homologous sequences. This results in the formation of the RAD51–ssDNA–dsDNA complex, leading to ssDNA strand invasion and D-loop formation [16–18]. We have previously shown that RAP1 and the basic domain of TRF2 (TRF2^B^) cooperate to prevent telomeric HDR [14, 15]. Cells lacking TRF2^B^ and functional RAP1 accumulate telomere D-loops, resulting in massive HDR-mediated telomere-telomere clustering and the formation of RAD51-dependent ultrabright telomeres (UTs) [15, 19].

Increasing evidence reveals that HDR requires active RNA transcription [20]. In the presence of DSBs, RNA transcripts form DNA–RNA R-loop hybrids in donor dsDNA, promoting strand invasion of ssDNA [21]. R-loop formation requires the RAD51 cofactor RADR51AP1 [22–24]. RAD51AP1-generated R-loops enhance RAD51-mediated formation of DNA–RNA (DR)-loops, structures that contain both DNA–DNA and DNA–RNA hybrids [21]. On telomeres, RAD51AP1 interacts with the long noncoding telomere repeat-containing RNA (TERRA) to form D- and R-loops to mediate telomere HDR [22, 23, 25]. While TERRA serves positive roles in promoting telomere HDR, it also represents a destabilizing force at telomeres, in that TERRA R-loops formed in *cis* can promote DNA breaks that negatively impact cell viability [26, 27]. Loss of dynamic regulation of telomere R-loops induces telomere instability, contributing to human disorders including ICF (Immunodeficiency, Centromeric instability, and Facial anomalies) syndrome and certain cancers [28–30]. Therefore, how TERRA R-loops are regulated at telomeres represents an important biological question.

We have previously shown that TRF2^B^ cooperates with RAP1 to repress TERRA R-loop-mediated formation of UTs [15]. UTs generated in *Rap1^−/−^* cells expressing TRF2^ΔB^ are reminiscent of the telomere clustering observed in the alternative lengthening of telomeres (ALT) pathway, a telomerase-independent, HDR-dependent mechanism to extend telomeres in a subset of human cancers [31]. ALT-associated PML bodies (APBs), a hallmark of ALT, were detected on UTs [15]. Since the UTs formed in the absence of TRF2^B^ and RAP1 are also found in telomerase-positive cells, UTs are likely not APBs but represent telomere–telomere HDR intermediates. Mammalian ALT occurs through both RAD52-dependent breakage-induced replication and RAD52-independent pathways [32].

Both RAD51 (with RAD51AP1) and RAD52 are able to catalyze the strand formation step in HDR to initiate ALT [33]. We found that TRF2’s interactions with ADAR1p110 and DDX21, two proteins that process RNA–DNA hybrids, are essential to repress UT formation [15]. Since overexpression of RNaseH1 and ADAR1p110 (but not their catalytically inactive mutants) abolished UT formation, our results suggest that TRF2^B^ and RAP1 recruit these proteins to telomeres to resolve R-loops [15]. However, how the TRF2–RAP1 complex represses TERRA-mediated telomeric R-loop formation and telomere HDR remains unclear.

Using reconstituted biochemical systems, we show that RAD51AP1 and TERRA-dependent R-loops promote RAD51-mediated telomere DR-loop formation. Both the TRF2–RAP1 complex and TRF2’s interaction with the BLM helicase are required to promote BLM-mediated unwinding of telomere R-loops. Importantly, BLM-deficient cells and cells expressing BLM mutants unable to interact with TRF2 accumulate telomere R-loops on UTs. Our findings highlight a novel mechanism in which the TRF2–RAP1–BLM complex promotes R-loop removal from telomeres to inhibit telomere D-loop formation, thus repressing telomere HDR and UT formation in cells.

## Materials and methods

### Protein expression and purification

#### TRF2, RAP1, BLM, and mutants

hRAP1 complementary DNA (cDNA) with a C-terminal Flag-tag was subcloned into the BglII/HindIII site of pTric-HisB vector. hTRF2 (Addgene #50488) or hRAP1 in pTricHisB vectors was transformed into *Escherichia coli* BL21(DE3) cells and purified by Ni Sepharose and anti-FLAG M2 affinity chromatography as described [19]. hBLM cDNA with a C-terminal Flag tag from pcDNA3/BLM (Addgene #111766) was subcloned into phCMV1-2 × MBP-MCS vector [19, 34]. TRF2 and BLM mutations were generated with a site-directed mutagenesis kit (Agilent). The phCMV1 construct with 2 × MBP-tagged hBLM was expressed in 293T cells using the calcium phosphate method and purified as previously described [19, 35].

#### RAD51AP1

hRAD51AP1 (isoform 2) harboring N-terminal Maltose-Binding Protein (MBP) and C-terminal His6 affinity tags were transformed into *Escherichia coli* BL21(DE3) cells. The cells were grown in Luria broth medium at 37°C until the OD_600_ reached 0.6. IPTG (final 0.4 mM) was used to induce protein expression at 37°C for 4 h and cells were harvested by centrifugation at 5000 rpm for 10 min. All the purification steps were conducted at 4°C. About 5 g cell pellet was resuspended in 20 ml cell breaking buffer [20 mM Tris–HCl, pH 7.5, 10% sucrose, 0.5 mM ethylenediaminetetraacetic acid (EDTA), 300 mM KCl, 0.1% Igepal CA-630, 1 mM DTT, 1 mM PSMF, and a cocktail of protease inhibitors (Roche)] and subject to sonication to prepare crude lysate. After centrifugation (14 000 × *g*, 60 min), the clarified lysate was mixed with 2 ml Ni Sepharose 6 Fast Flow (GE Healthcare) for 3 h. The matrix was poured into a column and sequentially washed twice with 50 ml buffer K (20 mM Tris–HCl, pH 7.5, 10% glycerol, 0.5 mM EDTA, 0.01% Igepal, 1 mM DTT) containing 1000 mM KCl or 300 mM KCl with 10 mM imidazole. RAD51AP1 proteins were eluted in 1 ml of 500 mM imidazole in buffer K containing 300 mM KCl three times. The eluates were combined, buffer K containing 300 mM KCl was added to 10 ml eluates and incubated with 1 ml of amylose resin for 16 h. After sequentially washing with 50 ml buffer K containing 1000 mM KCl or 300 mM KCl, the proteins were then eluted with buffer K with 300 mM KCl and 50 mM maltose. The eluate was concentrated by a Centricon-30K device (Millipore) to ~5 mg/ml and stored in small aliquots at −80°C. hRAD51 was expressed in *E. coli* and purified as described previously [19, 36].

#### Telomere DNA and TERRA substrates

The telomere ssDNA oligo (Tel90) containing (TTAGGG)_14_ repeats and the plasmid containing telomere sequences (TTAGGG)_17_ (PBB, Addgene #53210) were used in the D-loop assay. Telomere ssRNA oligo (TERRA) containing (UUAGGG)_5_ repeats was used in the R-loop formation assay. Telomere ssDNA (Tel90) with 5′-IRDye700 and TERRA with 5′-IRDye800CWN were used in the telomere D/R-loop assay. 5′-end biotin-labeled telomere ssDNA oligo (Biotin-Tel90) was used in the RAD51/telomere ssDNA filament stabilization and dsDNA/R-loop capture assays. The telomere dsDNA in capture assay was generated by annealing TDR1 and 5′ ^32^P-labeled oligonucleotide TDR2. The telomere R-loop in capture assay was generated by annealing TDR3, TERRA, and 5′ ^32^P-labeled oligonucleotide TDR2. The telomere R-loop substrate in unwinding assay was generated by hybridizing 5′ ^32^P-labeled TERRA to oligonucleotides TDR2 and TDR3.

The oligonucleotides used in this study were obtained from IDT and their sequences are listed in Supplementary Table S1.

#### *In vitro* telomeric transcription

Two micrograms of pcDNA6A-Telo1.6-Rev plasmid containing ∼1 kb of CCCTAA telomere repeats [37] were linearized with AgeI restriction enzyme and purified with QIAGEN DNA Clean & Concentrator kits. One microgram of linearized DNA was incubated with ATP, GTP, CTP, UTP (100 nM each), 2 μl of T7 RNA pol mix (NEB), and 2 μl of 10× T7 RNA pol buffer (NEB) in total 20 μl reaction for 4 h at 37°C. Then 140 μl of RNase-free H_2_O was added and incubated at 95°C for 5 min and quickly quenched on ice. To remove DNA template, 20 μl of buffer RDD and 10 μl of DNaseI (Qiagen) were added and incubated at 25°C for 1 h. RNeasy CleanUp Kit (Qiagen) was used to purify the RNA product.

#### Electrophoresis mobility shift assay

Purified TRF2 with or without RAP1 was incubated with 5′ ^32^P-labeled TERRA or R-loop (10 nM) in 10 μl reaction buffer D [25 mM Tris–HCl (pH 7.5), 50 mM KCl, 1 mM DTT, 100 mg/ml BSA, and 1.5 mM MgCl_2_] at 37°C for 10 min. The reaction mixtures were resolved in 10% polyacrylamide gels in 1× Tris-borate-EDTA (TBE) buffer [40 mM Tris–HCl (pH 8.3), 45 mM boric acid, and 1 mM EDTA] at 4°C. After drying, the radiolabeled RNA species were visualized by phosphorimaging.

#### Telomere D/R-loop assay

The D-loop assay was conducted as described previously [19, 24] with modifications. Briefly, ^32^P-labeled telomere ssDNA (Tel90) (25 nM) and RAD51 (0.8 μM) were pre-incubated in 10.5 μl of reaction buffer M (35 mM Tris, pH 7.5, 1 mM DTT, 5 mM MgCl_2_, and 50 mM KCl containing 2 mM ATP) at 37°C for 10 min. RAD51AP1 (100 nM) was pre-incubated with TERRA (10–40 nM) in buffer M at 37°C for 10 min. The plasmid PBB containing telomere sequence (TTAGGG)_17_ (35 μM) was added to the reactions and incubated at 37°C for 20 min. The reaction was deproteinized by proteinase K and SDS, followed by 1% agarose gel electrophoresis and phosphorimaging analysis. For the D/R-loop assay, 5′-IRDye700-labeled telomere ssDNA (Tel90) and 5′-IRDye800CWN-labeled TERRA were used, and electrophoresis results were analyzed by the ChemiDoc MP imaging system.

#### RAD51-telomere ssDNA filament stabilization assay

This assay was conducted as described previously [19, 38] with modifications. Briefly, 4 μl of streptavidin resin immobilized with 5′-biotinylated oligo Tel90 (10 μM) was incubated with RAD51 (2.7 μM) in 18 μl of reaction buffer A (40 mM Tris–HCl, pH 7.5, 50 mM KCl, 2 mM ATP, 5 mM MgCl_2_, 100 μg/ml BSA, and 1 mM DTT) containing an ATP-regenerating system consisting of 20 μM creatine phosphate and 20 μg/ml creatine kinase for 10 min at 37°C. TERRA (10–40 nM) and/or RAD51AP1 (100 or 400 nM) was added and incubated at 37°C for 20 min. Excess non-biotinylated oligo Tel90 (100 μM) was added in the reaction as a protein trap, and the reaction was mixed gently for 20 min at 37°C. The resin was centrifuged and washed twice, and then the bound proteins were eluted with 20 μl of 2% SDS. The 10% sodium dodecyl sulfate–polyacrylamide gel electrophoresis (SDS–PAGE), in which the supernatant fraction and the SDS eluate have been resolved by electrophoresis were Coomassie blue stained to determine RAD51 protein content.

#### Telomere duplex DNA and R-loop capture assay

This assay was conducted as described previously [19, 24] with modifications. RAD51 protein (3 μM) was incubated with streptavidin resin immobilized with 5′-biotinylated oligo Tel90 (10 μM) in 20 μl of reaction buffer W (35 mM Tris–HCl, pH 7.5, 50 mM KCl, 2 mM ATP, 1 mM MgCl_2_, 5 mM CaCl_2_, 100 μg/ml BSA, and 1 mM DTT) at 37°C for 10 min. The resin was centrifuged at 2000 rpm for 2 min and washed once with buffer W. The ^32^P radiolabeled telomere dsDNA or R-loop (4 μM) was added to the resin and incubated at 37°C for 20 min. The resin was washed twice, the bound proteins and radiolabeled DNA/R-loop were eluted with 20 μl of 2% SDS. The 10% native polyacrylamide gels in which the supernatant and SDS eluate have been resolved were dried and subjected to phosphorimaging analysis to reveal and quantify the radiolabeled dsDNA/R-loop.

#### Telomere D/R-loop unwinding assay

The telomere R-loop unwinding assay was performed in buffer B [20 mM Na-HEPES (pH 7.5), 2 mM ATP, 0.1 mM DTT, 100 mg/ml BSA, 0.05% Triton X-100, 2 mM MgCl_2_, and 100 mM KCl] and an ATP-regenerating system consisting of 20 mM creatine phosphate and 30 μg/ml creatine kinase. TRF2, RAP1, or the combination of both proteins (40 or 80 nM) was pre-incubated with telomere R-loop substrate (2.5 nM) on ice for 10 min. Then BLM (20 nM) was added and incubated at 37°C for 20 min. Reaction mixtures were deproteinized and analyzed by electrophoresis in 10% native polyacrylamide gels.

RAD51, RAD51AP1, 5′-IRDye700-labeled telomere ssDNA (Tel90), 5′-IRDye800CWN-labeled TERRA, and plasmid PBB were used to generate telomere D/R-loop. The deproteinized reaction mixtures by SDS and proteinase K were passed through Micro Bio-Spin 6 Column (Bio-Rad), equilibrated with buffer B. TRF2–RAP1 (50 nM) was pre-incubated with the D/R-loop substrate (2.5 nM) on ice for 10 min. Then BLM (20–80 nM) was added and incubated at 37°C for 20 min. Reaction mixtures were deproteinized and analyzed as described in the D/R-loop assay.

#### Cell culture, transfection, and Western blotting

The 3′-UTR shBLM with target sequence 5′- GAATCTCAATGTACATAGA −3′ was cloned into the pSUPER.retro.puro vector (Clontech) and transfected into 293T cells by LipoD293 (SignaGen) to generate shBLM retroviral supernatants. The HA-tagged cDNAs coding for the WT and mutant forms of BLM were introduced into the pQCXIP vector (Clontech). The generation of shTRF2 and TRF2^ΔB, L288R^ mutants was described previously [15, 19]. DNA constructs for shTRF2, shBLM, and Myc-TRF2^ΔB; L288R^ were transfected into 293T cells using Fugene 6 and packaged into retroviral particles. Viral supernatants were collected 48–72 h after transfection, filtered with 0.45 μm Millex filter, and directly used to infect U2OS cells. Transient transfections of HA-tagged BLM were performed using Fugene 6. Cells were lysed in urea lysis buffer (8 M urea, 50 mM Tris–HCl, pH 7.4, and 150 mM ß-mercaptoethanol) and protein concentration was determined using the Bradford assay (Bio-Rad). The proteins were resolved on 4%–12% SDS–PAGE gel.

For immunoblotting, the following antibodies were used: α-TRF2 (Millipore, #05‐521), α-Myc (Millipore, #05‐724), α-BLM (Santa Cruz, sc-365753), α-HA (Sigma, #H3663), and α-Tubulin (Fisher, # A11126). The ChemiDoc MP imaging system was used to document the chemiluminescence Western blots.

#### Immunofluorescence microscopy and fluorescent *in situ* hybridization

Cells grown on coverslips were infected and transfected with shRNAs or cDNAs. Cells were washed with cold PBS buffer and fixed in 100% ice-cold methanol for 7 min at −20°C followed by PBS washes. Cells on coverslips were blocked in PBG blocking solution (0.2% fish gelatin and 0.5% BSA in PBS) overnight at 4°C. Then cells were incubated with anti-DNA–RNA Hybrid (S9.6, Kerafast #ENH001) overnight at 4°C. After PBS washes, cells were incubated with Alexa Fluor 488 mouse secondary antibody for 1 h at room temperature. After washing with PBS, the cells were hybridized with TelC-Cy3 (CCCTAA)_3_ PNA telomere probe (PNA Bio, F1002) in hybridization buffer (0.5 μg/ml tRNA, 1 mg/ml BSA, 0.06× SSC, 70% formamide) at room temperature overnight in a humid chamber. After washing, the cells on coverslips were mounted with antifade mounting medium (Vectashield # H1200) with DAPI. Ultrabright telomeres (UTs) are defined as telomeric foci greater than 250 arbitrary units in size after a 10-millisecond exposure with an Andore CCD camera (chip bin setting 1), representing >8-fold increase in average normal telomere foci size [15]. Image acquisition and quantification of the size (area of telomeric fluorescent signals) of UTs were performed using a NIS-Elements BR (Nikon) system.

#### Statistical analysis

For our biochemistry assays, the signal intensity in gel bands was quantified using Quantity One (Bio-Rad). For our UT and R-loop colocalization assay, a minimum 200 nuclei were analyzed. Statistical analysis was conducted by GraphPad Prism 9 software and statistical evaluation was performed by one-way ANOVA or unpaired t test. ns: non-significant (*P* ≥ 0.05); *: 0.01 < *P *< 0.05; **: 0.001 < *P *< 0.01; ***: 0.0001 < *P *< 0.001; ****: *P *< 0.0001.

## Results

### RAD51AP1 and TERRA promote RAD51-mediated telomere D-loop formation

We have shown previously that cells lacking functional RAP1 and expressing TRF2^ΔB^ display massive telomere HDR, manifested as multiple telomere–telomere clustering into UTs [15]. UT formation requires the accumulation of RAD51-mediated D-loops on telomeres [19]. Several factors, including TERRA, influence D-loop formation and stability during telomere HDR [17, 21, 39]. TERRA has been shown to promote telomere HDR by promoting R-loop formation [25, 37]. However, the mechanism of how shelterin components influence TERRA’s ability to form telomere D-loops and telomere HDR remains unclear.

**Figure 1.**
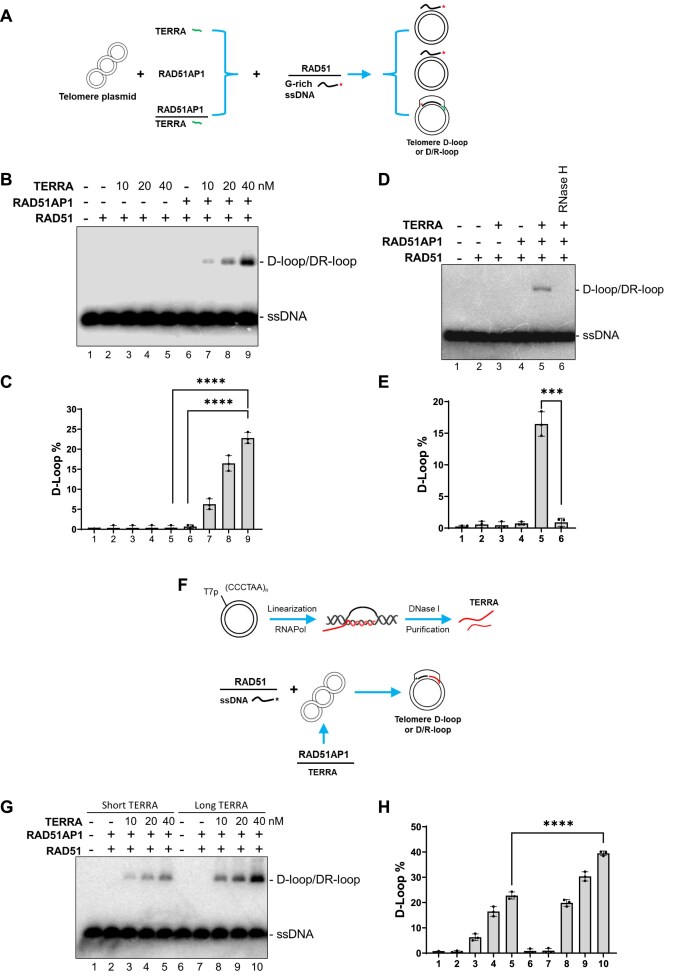
RAD51AP1 and TERRA promote RAD51-mediated telomeric D-loop formation. (**A**) Schematic of the RAD51/ssDNA and RAD51AP1/TERRA-induced telomere D-loop assay. ^32^P-labeled (asterisk) telomere G-rich ssDNA was first incubated with purified RAD51 protein to form RAD51/ssDNA filaments. RAD51AP1 and/or TERRA (green) were incubated with telomere plasmids and then mixed with preformed RAD51/ssDNA filaments. RAD51/ssDNA-mediated strand invasion into the telomere plasmids formed telomeric D-loops. (**B**) RAD51AP1 and TERRA promote RAD51-mediated telomere D-loop formation. RAD51AP1 protein (100 nM), TERRA (10, 20, 40 nM), and the indicated combinations were tested for their ability to promote telomere D-loop formation. D-loop and D/R-loop formation were determined using 1% agarose gel electrophoresis. (**C**) Quantification of the percentages of RAD51-mediated telomere D-loops in panel (B) is shown as mean ± SD from three independent experiments. ANOVA test was used to evaluate statistical difference. **** *P* < .0001. (**D**) RAD51-mediated telomere D-loop formation is DNA/RNA hybrid dependent. RAD51/ssDNA, RAD51AP1, and TERRA-induced telomere D-loop formation were examined. In lane 6, the sample was treated with RNase H (0.5 unit) for 10 min before adding RAD51/ssDNA filament. D-loop and D/R-loop formation were determined using 1% agarose gel electrophoresis. (**E**) Quantification of data from panel (D) showing the percentages of telomere D-loops before and after RNase H treatment displayed as mean ± SD from three independent experiments. Statistical evaluation was performed by ANOVA test. *** *P* = .00019. (**F**) Schematic showing the generation of long TERRA by *in vitro* transcription. ∼1 kb-long TERRA transcripts were obtained from pcDNA6A-Telo1.6-Rev plasmids via *in vitro* transcription using T7 RNA polymerase. Long TERRA transcripts were used in RAD51AP1/ long TERRA-induced, RAD51/ssDNA-dependent telomere D-loop assays. (**G**) RAD51AP1 and long TERRA promote RAD51/G-rich ssDNA-mediated telomere D-loop formation more efficiently than short TERRA. RAD51AP1, short or long TERRA, and the indicated combinations were tested for their ability to promote RAD51-mediated telomeric D-loop formation using 1% agarose gel electrophoresis. (**H**) The percentages of RAD51-mediated telomere D-loop formation by short and long TERRA and RAD51AP1 in panel (G) are shown as mean ± SD from three independent experiments. Statistical evaluation was performed by ANOVA test. **** *P* < .0001.

To address this question, we examined the ability of purified human RAD51 protein, G-rich telomeric ssDNA, and TERRA containing (UUAGGG)_5_ repeats to form D-loops in the presence of a telomere-repeat-containing donor plasmid. We used a well-established *in vitro* telomere D-loop assay (Fig. 1A) [19, 40], in which the presence of Mg^2+^ abolished ATP-dependent RAD51–ATP–ssDNA filament formation [36, 41]. In this condition, RAD51 by itself was unable to form D-loops on telomeres (Fig. 1B, lane 2). The addition of TERRA or RAD51AP1 together with RAD51 also does not promote RAD51-mediated D-loop formation (Fig. 1A, 1B, lanes 3–6). However, the addition of TERRA, RAD51AP1, and RAD51 together with the telomere plasmid significantly enhanced RAD51-mediated telomere D-loop formation even in the presence of Mg^2+^ (Fig. 1B, lanes 7–9; Fig. 1C).

Previous reports show that RAD51AP1 itself was unable to form D-loops but directly bound TERRA to stimulate RAD51-dependent D-loop formation [21–23]. Given that RNA transcripts form DNA–RNA hybrids in donor DNA to promote invasion of ssDNA to form DNA–RNA hybrid DR-loops and increased HDR [21], we asked whether the enhancement of telomere D-loop formation by both RAD51AP1 and TERRA might be due to the generation of DR-loops. To test this hypothesis, RAD51AP1 and TERRA were first pre-incubated with the telomere donor plasmid, and then RNase H was added before addition of RAD51/ssDNA filament. We discovered that RNase H treatment eliminated D-loop formation (Fig. 1D, compare lanes 5 and 6; Fig. 1E). This result suggests that formation of telomere R-loops is required for the generation of telomere D-loops.

Having established that short TERRA repeats efficiently generated telomere D-loops, we next examined whether longer TERRA transcripts increased the efficiency of telomere D-loop formation. We used telomere plasmids containing ~1 kb of CCCTAA repeats [37] and used *in vitro* transcription to generate long G-rich TERRA transcripts (Fig. 1F). We found that long G-rich TERRA transcripts generated significantly more D-loops than the short G-rich TERRA (Fig. 1G, compare lanes 3–5 with lanes 8–10; Fig. 1H). Taken together, our data suggest that long G-rich TERRA together with RAD51AP1 efficiently promoted RAD51-mediated telomere D-loop formation in a DR loop-dependent manner.

### The RAD51/ssDNA filament captures telomere R-loops preferentially over dsDNA

**Figure 2.**
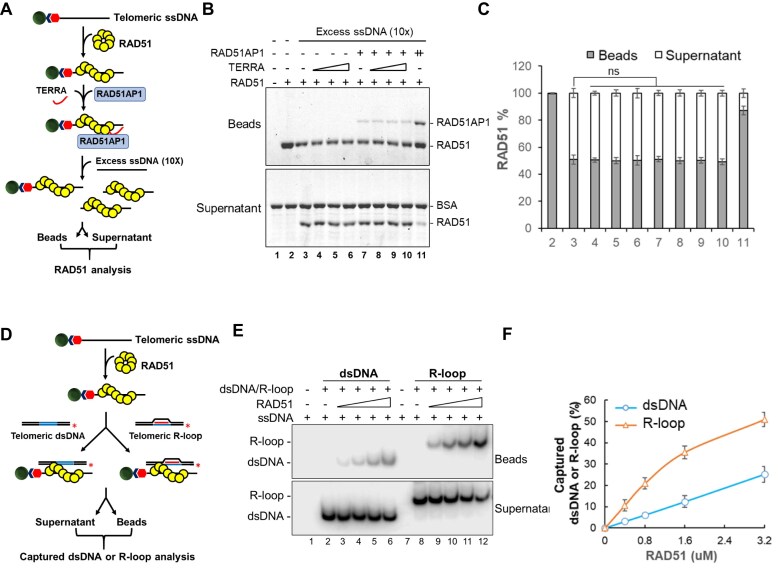
RAD51/ssDNA filament captures telomere R-loop preferentially over dsDNA. (**A**) Schematic of the assay used to examine the effect of RAD51AP1 or TERRA on the stability of RAD51-coated telomeric ssDNA filaments. RAD51 protein was used to assemble filaments on 5′-biotinylated telomeric ssDNA that was immobilized on streptavidin resin. RAD51AP1 protein and/or TERRA was incubated with the RAD51-coated telomere ssDNA filaments and then non-biotinylated telomeric ssDNA at 10× excess was added to the reaction. Both the bead fractions containing proteins associated with the biotinylated ssDNA and the supernatant fractions containing proteins trapped on the excess ssDNA were analyzed by SDS–PAGE with Coomassie Blue staining. (**B**) TERRA and RAD51AP1 do not affect RAD51 filament stabilization on telomere ssDNA. TERRA (10, 20, 40 nM) and RAD51AP1 (100 nM) either individually or in combination were tested for RAD51-mediated telomere ssDNA filament stabilization. A higher concentration of RAD51AP1 (400 nM, lane 11) promotes RAD51/ssDNA filament stabilization. (**C**) Quantification of the percentages of RAD51 protein in the supernatant and bead fractions, plotted as mean ± SD from two independent experiments. Statistical evaluation was performed by unpaired t-test analysis. ns, non-significant (*P* > .05). (**D**) Schematic of the telomere dsDNA or R-loop capture assays by RAD51 filaments. RAD51 was used to assemble filaments on 5′-biotinylated telomere ssDNA that was immobilized on streptavidin resin, and then these filaments were incubated with either ^32^P-labeled telomere dsDNA or telomere R-loops. Radiolabeled dsDNA or R-loops on the bead and supernatant fractions were resolved by electrophoresis in 10% native polyacrylamide gels. (**E**) RAD51-bound telomere ssDNA filaments more efficiently capture telomere R-loops over dsDNA. RAD51 (0.4, 0.8, 1.6, 3.2 μM)-coated telomere ssDNA (10 μM) filaments were incubated with telomere dsDNA or R-loops (4 μM), and their ability to capture telomere dsDNA or R-loops was analyzed by electrophoresis in 10% native polyacrylamide gels. (**F**) Quantification of the percentages of captured telomere dsDNA or R-loops in the bead fractions. The error bars represent mean values ± SD of data from three independent experiments.

Given that both RAD51AP1 and TERRA interact with RAD51 [16, 25], we next asked whether they might help stabilize RAD51–ssDNA nucleofilaments, a prerequisite for D-loop formation on telomeres. We first assembled RAD51 on telomere ssDNA and then challenged this complex with 10× excess of telomeric ssDNA (Fig. 2A). In the absence of excess telomeric ssDNA, RAD51 stably bound telomeric ssDNA and localized to the bead fraction (Fig. 2B, lane 2). Approximately 50% of RAD51 was found in the supernatant in the presence of excess telomere ssDNA (Fig. 2B, lane 3; Fig. 2C). Under these conditions, the addition of increasing concentrations of TERRA either alone or in combination with 100 nM of RAD51AP1 had no effect on RAD51 filament stabilization (Fig. 2B, lanes 4–10; Fig. 2C). As a positive control, we show that at high concentration, RAD51AP1 promoted RAD51-telomeric ssDNA filament stabilization (Fig. 2B, lane 11; Fig. 2C). Our results suggest that the addition of TERRA or RAD51AP1 does not impact stably formed RAD51-telomere ssDNA complexes.

We have previously shown that the stable RAD51–ssDNA nucleoprotein filaments on telomeres possess the ability to capture telomere dsDNA to perform homology search to form D-loops [19]. We next asked whether the RAD51-telomere ssDNA filaments preferred to interact with telomere R-loops over telomere dsDNA. To address this question, we assembled RAD51 filaments on 5′-biotinylated telomere ssDNA immobilized on streptavidin resin and then incubated them with either radiolabeled telomere dsDNA or telomere R-loops. Biotin-tagged telomere ssDNA bound to either dsDNA or R-loops was pulled down on SA-coated beads, followed by polyacrylamide gel electrophoresis to quantify the amount of radiolabeled telomere dsDNA or R-loops (Fig. 2D). We show that RAD51-telomere ssDNA filaments captured both telomere dsDNA (Fig. 2E, lanes 3–6) and telomere R-loops (Fig. 2E, lanes 9–12) in a concentration-dependent manner. Importantly, our data reveal that RAD51/ssDNA filaments captured telomere R-loops preferentially over telomere dsDNA (Fig. 2F).

### RAD51AP1 and TERRA-dependent R-loops enhance telomere D-loop formation

**Figure 3.**
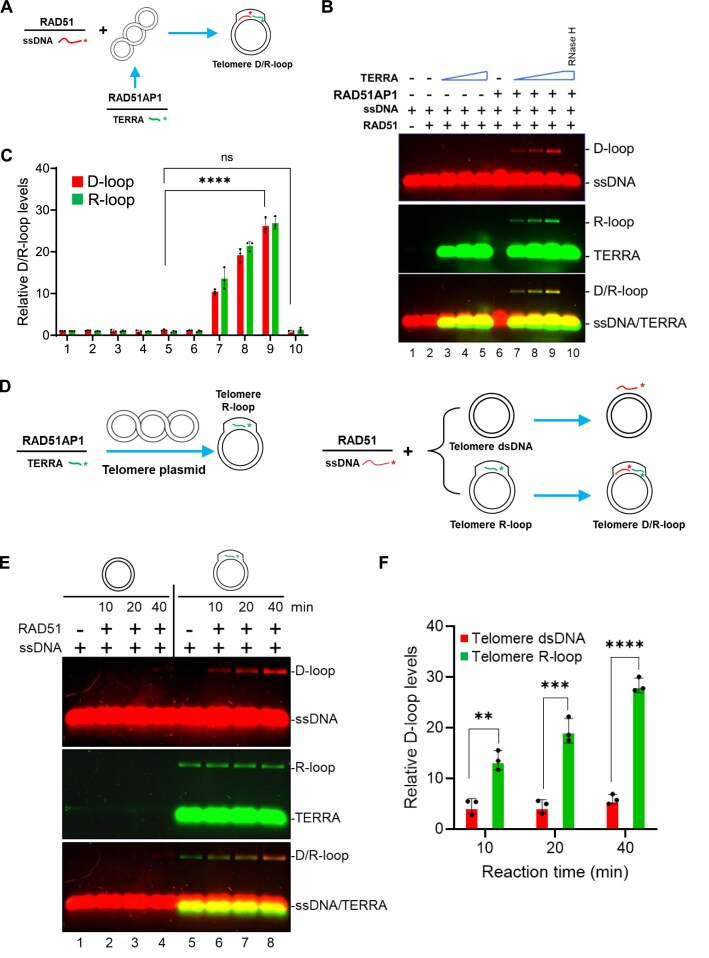
RAD51AP1 and TERRA-dependent R-loops enhance D-loop formation. (**A**) Schematic of the RAD51/ssDNA and RAD51AP1/TERRA-induced telomere D/R-loop assay. IRDye-700-labeled telomere G-rich ssDNA (red) was first incubated with purified RAD51 protein to form RAD51/ssDNA filaments. RAD51AP1 and/or IRDye-800-labeled TERRA (green) were incubated with telomere plasmid to form R-loops and then mixed with pre-formed RAD51/ssDNA filaments. ssDNA or TERRA invasion into the plasmid formed telomere D/R-loops. Red or green asterisks indicate the labeled ends of ssDNA or TERRA. (**B**) TERRA-R-loops enhance telomere D-loop formation. RAD51AP1, TERRA, and the indicated combinations were tested for their ability to enhance telomere D-loop formation. In lane 10, the sample was treated with RNase H (0.5 unit) for 10 min before adding RAD51/ssDNA filaments. D/R-loop formation (yellow signal) was visualized by 1% agarose gel electrophoresis. (**C**) Quantification of the percentages of relative D-loops and R-loops. The error bars represent mean values ± SD of data from three independent experiments. Statistical evaluation was performed by ANOVA. ns: non-significant (*P *= .1758); **** *P* < .0001. (**D**) Schematic of the assay used to measure the ability of RAD51/ssDNA filament to form D-loops on telomere dsDNA and telomere R-loops. Left panel: Telomere R-loops were generated by incubating RAD51AP1, IRDye-800-labeled TERRA (green), and a plasmid containing telomere repeats together. Native plasmid-sized telomere R-loops were obtained after deproteinization and column purification. Right panel: To generate telomeric D/R loops, IRDye-700-labeled telomere ssDNA (red) was first incubated with purified RAD51 protein to form RAD51/ssDNA filaments and then incubated with telomere dsDNA or telomere R-loops. D/R-loop formation (yellow signal) was visualized by 1% agarose gel electrophoresis. (**E**) Telomere R-loops promote D/R-loop formation. RAD51 and telomere ssDNA filaments were incubated with telomere dsDNA or R-loops for the indicated times, and telomere D/R-loop formation (yellow signal) was analyzed by 1% agarose gel electrophoresis. (**F**) Quantification of D-loop formation in the presence or absence of telomere R-loops. The amounts of telomere D-loops formed relative to the negative control (no RAD51, lanes 1 and 5) on telomere dsDNA or R-loops were quantified and plotted as mean ± SD from three independent experiments. Statistical evaluation was performed by ANOVA test. ** *P *= .002956; *** *P* = .0007408; **** *P* < .0001.

In the genome, local RNA transcripts promote DSB repair by initiating HDR, and R-loops stimulate local D-loop formation by forming DR-loops *in vitro* [21]. To more clearly define how telomere D-loop formation is enhanced by DR-loops, we labeled telomere ssDNA (TTAGGG)_14_ with IRDye700 (red) and TERRA containing (UUAGGG)_5_ repeats with IRDye800CWN (green) and subjected them to telomere D-loop assays (Fig. 3A). RAD51AP1 and IRDye800CWN-TERRA were first pre-incubated with telomere plasmids. Pre-formed RAD51 filaments on IRDye700-telomere ssDNA were then added into the reaction to test for D-loop and R-loop formation. RAD51AP1 and TERRA induced telomere R-loop formation, and consistent with our findings in Fig. 1, telomere D-loops formed only in the presence of both TERRA and RAD51AP1 (Fig. 3B, lanes 7–9; Fig. 3C). The merged images of D-loops (red) with R-loops (green) yielded a yellow band, indicating the formation of D/R-loops. RNase H treatment before incubating the RAD51/ssDNA filaments with the telomere plasmid abolished both R-loop and D-loop formation (Fig. 3B, lane 10), confirming that telomere R-loop formation is required for the generation of telomere D-loops. In contrast, RNase H treatment after D/R-loop formation abolished R-loop formation, while D-loop formation remained unaffected (Supplementary Fig. S1A and B).

After demonstrating that RAD51 filaments on telomeric ssDNA preferred to capture telomere R-loops over telomere dsDNA (Fig. 2E and F), we proceeded to test the hypothesis that RAD51/ssDNA filaments more favorably form D-loops on RAD51AP1-TERRA R-loops over telomere dsDNA. Telomere R-loops were generated by incubating RAD51AP1 with IRDye800CWN-TERRA and telomere plasmids. Native telomere R-loops were obtained after deproteinization (Fig. 3D, left panel). Telomere plasmids with or without R-loops were used to test D-loop formation by exposure to RAD51/IRDye700-telomere ssDNA filaments (Fig. 3D, right panel). We found that compared to telomere dsDNA substrates, RAD51/ssDNA filaments formed D-loops ∼5-fold higher on telomere R-loop substrates, preferentially generating D/R-loops (Fig. 3E, lane 8; Fig. 3F).

### TRF2–RAP1 interaction enhances TRF2 binding to TERRA R-loops

**Figure 4.**
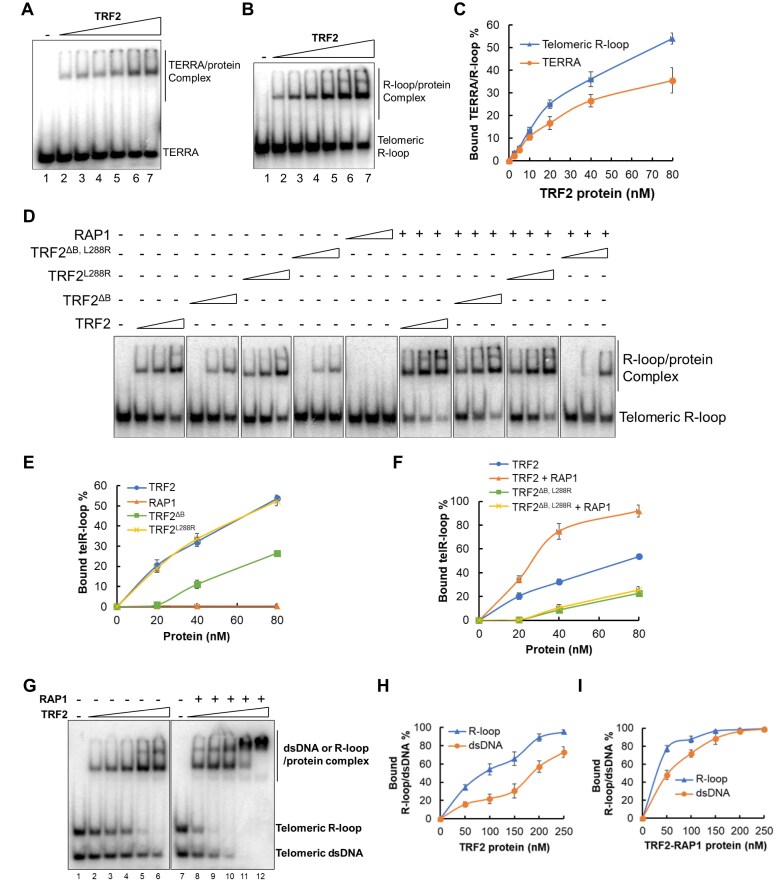
TRF2–RAP1 interaction enhances TRF2’s binding to telomere R-loops. (**A, B**) TRF2 binds ^32^P-TERRA and telomere R-loops. His-tagged TRF2 protein (0–80 nM) was incubated with 5 nM radiolabeled TERRA (A) or telomere R-loops (B). The mobility shifts of the TRF2–RNA complex were analyzed by 10% native polyacrylamide gel electrophoresis. (**C**) Quantification of the binding data in panels (A, B). The error bars represent mean values ± SD of data from three independent experiments. (**D**) TRF2–RAP1 interaction enhances TRF2’s binding to telomere R-loops. Purified WT TRF2, mutants TRF2^ΔB^, TRF2^L288R^, TRF2^ΔB,L288R^, and WT RAP1 alone or in the indicated combinations were tested for telomere R-loop binding. The mobility shift of the TRF2–RNA complexes was analyzed by 10% polyacrylamide gels. (**E, F**) Quantification of the R-loop binding data in panel (D). Error bars represent mean values ± SD of data from three independent experiments. (**G**) TRF2 (50, 100, 150, 200, and 250 nM) without or with RAP1 (100 nM) was incubated with telomere dsDNA and R-loops (10 nM each) to determine relative binding affinities. The ability of TRF2 or TRF2–RAP1 to bind to these nucleic acid substrates was analyzed by 10% polyacrylamide gels. (**H, I**) The R-loop and dsDNA binding data in panel (G) were quantified and plotted. Error bars represent mean values ± SD of data from three independent experiments.

*RAP1^−/−^* cells expressing TRF2^ΔB^ accumulate R-loops on clustered telomeres [15]. To test whether TRF2 binds TERRA and R-loops, the mobility shift of radiolabeled TERRA or R-loops in the presence of TRF2 was examined. Our results show that TRF2 efficiently bound both types of RNA substrates (Fig. 4A–C). The TRF2^ΔB^ mutant bound R-loops two-fold less efficiently than WT TRF2, whereas the TRF2 mutant defective in RAP1 binding (TRF2^L288R^) bound R-loops as well as WT TRF2 (Fig. 4D and E). While RAP1 does not bind telomere R-loops, the TRF2–RAP1 complex displayed a three-fold increase in R-loop binding activity compared to WT TRF2 alone (Fig. 4D and F). This increased ability to bind R-loops requires the formation of the TRF2–RAP1 complex, since neither the TRF2^L288R^ mutant that cannot interact with RAP1, nor the TRF2^ΔB, L288R^ double mutant bound R-loops as tightly as the TRF2–RAP1 complex (Fig. 4D and F).

Finally, we tested the relative affinities of TRF2 and TRF2–RAP1 for telomere R-loops versus dsDNA. Co-incubation of TRF2 with both nucleic acid substrates revealed a distinct preference of TRF2 for R-loops (Fig. 4G and H). Compared to TRF2 alone, the addition of RAP1 to TRF2 increased the binding affinities of the complex to both R-loops and dsDNA, with a distinct preference of TRF2–RAP1 for R-loops over dsDNA (Fig. 4G and I).

### TRF2–RAP1 promotes BLM-mediated unwinding of telomere R-loops

**Figure 5.**
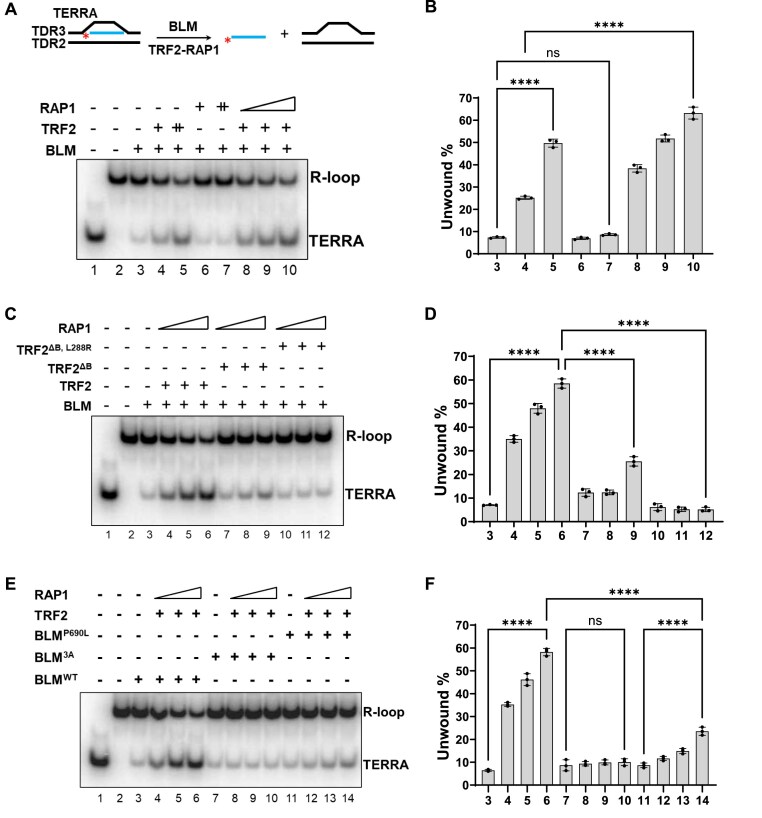
TRF2–RAP1 promotes BLM-mediated unwinding of telomere R-loops. (**A**) (Top) Schematic of the oligo-based telomere R-loop unwinding assay. Telomere R-loop substrates were generated by hybridizing ^32^P-labeled TERRA and two telomere DNA fragments (TDR2 and TDR3). TRF2 and/or RAP1 were pre-incubated with the R-loops and then BLM was added to the reaction, and the complex was resolved by 10% native polyacrylamide gel electrophoresis to monitor for R-loop unwinding. Displacement of the invading radiolabeled TERRA from R-loops indicates that R-loop unwinding. (Bottom) The TRF2–RAP1 complex promotes BLM-mediated unwinding of telomere R-loops. The effects of TRF2 alone (40, 80 nM) or in combination with RAP1 (20, 40, 80 nM) on the ability of BLM (20 nM) to unwind telomere R-loops were examined. ^32^P-labeled TERRA and R-loops were resolved by native-PAGE and shown in lanes 1 and 2. (**B**) Quantification of BLM-mediated R-loop unwinding reactions in panel (A). The percentages of unwound R-loops are shown as mean values ± SD from three independent experiments. Statistical evaluation was performed by ANOVA test. ns: non-significant (*P* = .9485); **** *P* < .0001. (**C**) The TRF2 basic domain is required for efficient unwinding of telomere R-loops. The effect of WT TRF2, TRF2^ΔB^, TRF2^ΔB,L288R^, and RAP1 to enhance BLM-mediated telomere R-loop unwinding was examined. The sizes of ^32^P-labeled TERRA and R-loops were resolved by native-PAGE, as shown in lanes 1 and 2. (**D**) Quantification of BLM-mediated R-loop unwinding reactions in panel (C). The percentages of unwound R-loops are shown as mean values ± SD from three independent experiments. Statistical evaluation was performed by ANOVA test. **** *P* < .0001. (**E**) The TRF2–BLM interaction enhances telomere R-loop unwinding. The effect of TRF2–RAP1 on the ability of WT and mutant BLM (3A or P690L) to unwind telomere R-loops was tested as in Fig. 5A. ^32^P-labeled TERRA and R-loops were loaded as size markers (lanes 1 and 2) and resolved by native-PAGE. (**F**) Quantification of the percentages of unwound R-loops in panel (E) as mean ± SD from three independent experiments. Statistical evaluation was performed by ANOVA test. ns: non-significant (*P* = .98); **** *P* < .0001.

The Bloom syndrome helicase (BLM) plays crucial roles in maintaining genome stability by disrupting RAD51–ssDNA filament formation and stimulating DNA synthesis during HDR [42, 43]. BLM also plays a critical role in telomere maintenance by participating in HDR-mediated alternative lengthening of telomeres (ALT) [44–47]. We and others have previously shown that the BLM helicase possesses telomere D-loop unwinding activity [19, 48] and that BLM-deficient cells accumulate telomere D-loops [19]. To test whether BLM also unwinds TERRA R-loops, an oligo-based TERRA R-loop structure was generated by annealing an invading radiolabeled TERRA containing (UUAGGG)_5_ with 2 DNA oligonucleotides containing telomere sequences. TRF2 and/or RAP1 were first pre-incubated with this R-loop substrate and then BLM was added to the reaction to detect R-loop unwinding activity. Displacement of the invading TERRA strand from the R-loops indicates R-loop unwinding (Fig. 5A). Consistent with previous findings that BLM unwinds DNA–RNA hybrids containing random oligonucleotides [49, 50], we found that BLM was able to unwind TERRA R-loops (Fig. 5A, lane 3). Importantly, BLM-mediated unwinding of TERRA R-loops was stimulated five-fold by the addition of TRF2, but not RAP1 (Fig. 5A, compare lanes 3 and 4–7; Fig. 5B). Compared to TRF2 alone, the TRF2–RAP1 complex significantly increased BLM’s ability to unwind TERRA R-loops (Fig. 5A, compare lanes 4 and 8–10; Fig. 5B). This enhancement of BLM-mediated TERRA R-loop unwinding required TRF2’s basic domain and the ability of TRF2 and RAP1 to form a complex, since both the TRF2^ΔB^ and TRF2^ΔB, L288R^ mutants were significantly less effective at stimulating BLM’s TERRA R-loop unwinding activity (Fig. 5C, compare lanes 4–6 with lanes 7–9, 10–12; Fig. 5D).

We and others have shown previously that BLM directly interacts with TRF2 [19, 48] and identified the BLM^3A^ and BLM^P690L^ mutants that are impaired in their interactions with TRF2 [19]. However, both BLM mutants retain their helicase activity to unwind telomere R-loops (Supplementary Fig. S2A and B). We next asked whether the enhancement of BLM-mediated TERRA R-loop resolution by TRF2–RAP1 is dependent on BLM’s ability to interact with TRF2. We show that both BLM^3A^ and BLM^P690L^ mutants failed to be stimulated by TRF2–RAP1 to unwind TERRA R-loops (Fig. 5E, compare lanes 4–6 and 8–10, 12–14; Fig. 5F). In addition, the BLM helicase activity is required for its ability to unwind telomere R-loops, because the helicase-dead BLM mutant (BLM^K695R^) failed to show unwinding activity even in the presence of TRF2–RAP1 (Supplementary Fig. S2C and D). These results reveal that the stimulatory effects of TRF2–RAP1 on BLM’s ability to unwind TERRA R-loops are dependent on TRF2’s ability to interact with BLM plus BLM’s own helicase activity.

### BLM preferentially releases TERRA over ssDNA from telomere D/R-loops

**Figure 6.**
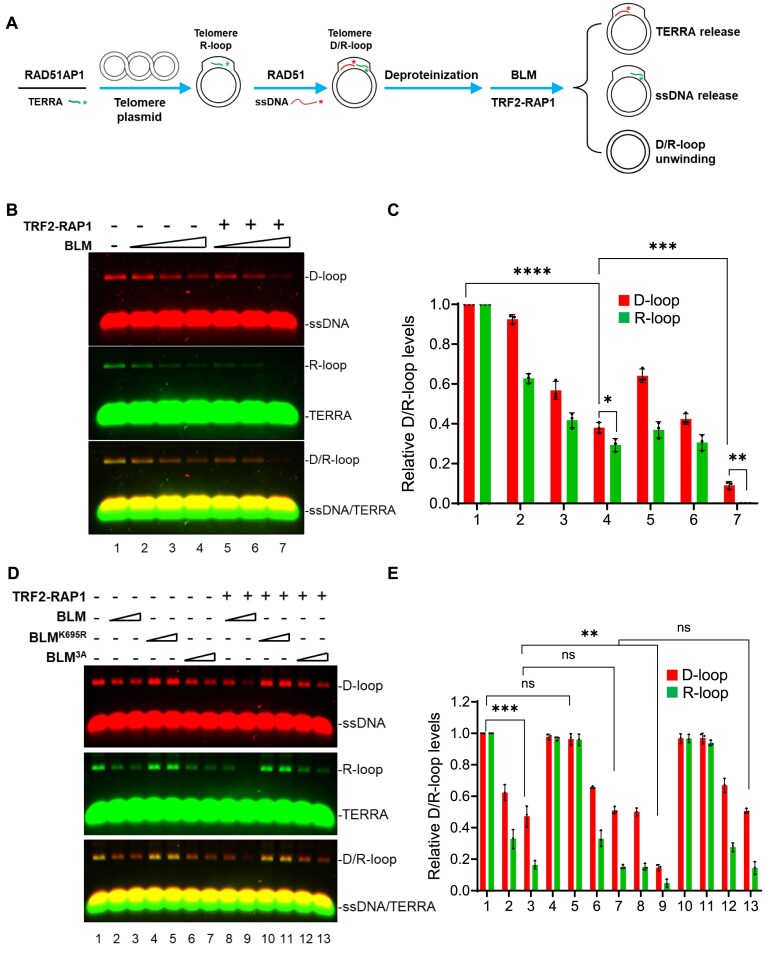
BLM preferentially releases TERRA over ssDNA from telomere D/R-loops. (**A**) Schematic of the assay used to measure how TRF2–RAP1 promotes BLM-mediated unwinding of RAD51/ssDNA and RAD51AP1/TERRA-generated telomeric D/R-loops. Telomere D/R-loops were generated by incubating RAD51 with IRDye-700-labeled telomere ssDNA (red), RAD51AP1 with IRDye-800-labeled TERRA (green), and telomere plasmids together as described in Fig. 3A. Native plasmid-sized telomere D/R-loops were obtained after deproteinization and column purification. BLM with or without TRF2–RAP1 was then incubated with these D/R-loops, and ssDNA, TERRA release, or D/R-loop unwinding was analyzed by 1% agarose gels. (**B**) BLM preferentially releases TERRA over ssDNA from telomere D/R-loop. BLM (20, 40, 80 nM) was tested for its ability to unwind telomere D/R-loops or TRF2–RAP1-bound D/R-loops. ssDNA, TERRA release, or D/R-loop unwinding was analyzed by 1% agarose gels. The unwinding of telomere D/R-loops by BLM was enhanced by TRF2–RAP1. (**C**) Quantification of the amount of D- and R-loops relative to the negative control (no proteins, lane 1). Data were plotted as mean ± SD from three independent experiments. Statistical evaluation was performed by ANOVA test. *P = .02282; **P = .001278; ***P = .0007284; **** *P* < .0001. (**D**) The effects of TRF2–RAP1 on WT BLM, the helicase-dead BLM^K695R^ or BLM^3A^ mutants on D/R-loop unwinding were tested as in panel (B). In contrast to WT BLM, TRF2–RAP1 cannot enhance BLM^3A^’s ability to unwind telomere D/R-loops. D/R-loop unwinding was analyzed by 1% agarose gels. (**E**) Quantification of the relative amounts of D-loops or R-loops to the control without proteins (lane 1) is shown as mean ± SD from three independent experiments. ANOVA test was used to evaluate statistical differences. ns: non-significant (*P* = .15; .4147; .8026); ** *P *= .001193; *** *P* = .000158.

Having established that the BLM helicase unwinds both telomere D-loops and TERRA R-loops, we next investigated how it releases TERRA and ssDNA from RAD51- and RAD51AP1-generated telomeric D/R-loops. RAD51AP1 and IRDye800CWN-TERRA were pre-incubated with the telomere plasmid, and then the pre-formed RAD51 filaments on IRDye700-telomere ssDNA were added into the reaction to generate telomeric DR-loops. After deproteinization and purification, the native telomere D/R-loops were incubated with BLM with or without TRF2–RAP1, and D/R-loop unwinding was analyzed (Fig. 6A). We found that BLM unwound both telomere D-loops and TERRA R-loops, and this activity increased in the presence of TRF2–RAP1 (Fig. 6B and C). Compared to telomere ssDNA, BLM released TERRA more efficiently from the telomere D/R-loops (Fig. 6B, lanes 2–4 to lanes 5–7 for D-loop, R-loop, and merged DR-loop formation; Fig. 6C). While the BLM helicase-dead mutant BLM^K695R^ failed to unwind telomere DR-loops, the BLM^3A^ mutant retained WT BLM’s telomeric DR-loop unwinding activity (Fig. 6D, lanes 2–7). Importantly, TRF2–RAP1 significantly enhanced WT BLM but not BLM^3A^ mutant’s ability to unwind telomere DR-loops (Fig. 6D, lanes 8–13; Fig. 6E). In addition, the TRF2–BLM interaction is required to promote BLM’s ability to unwind telomere D/R-loops. Collectively, our results provide strong evidence that the RAP1–TRF2–BLM complex is important to unwind telomere DR-loops, and this complex preferentially released TERRA over ssDNA from telomere D/R-loops.

### The RAP1–TRF2–BLM complex is required to resolve telomere R-loops in U2OS cells

**Figure 7.**
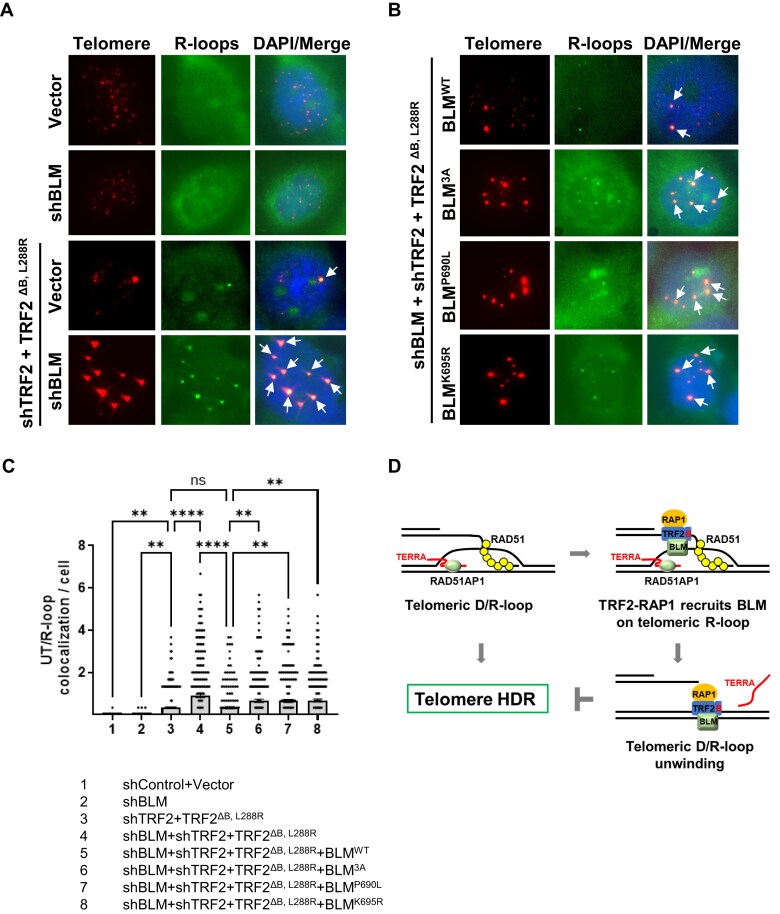
TRF2–RAP1–BLM is required to resolve telomere R-loops in U2OS cells. (**A**) U2OS cells expressing TRF2^ΔB, L288R^ were treated with shControl, shBLM, or shTRF2. Immunofluorescence-FISH analysis of cells containing UTs (PNA telomere probe, red) co-localized with R-loops (S9.6 antibody, green) and DAPI-stained nuclei (blue). White arrow: co-localization of R-loops on UTs. (**B**) U2OS cells expressing shBLM-resistant WT BLM cDNA and indicated BLM mutants were treated with shBLM, shTRF2, and TRF2^ΔB, L288R^. IF-FISH analysis was performed to detect UT/R-loop co-localization. White arrow: co-localization of R-loops on UTs. (**C**) Quantification of data from Fig. 6A and B, showing the number of UT/R-loop colocalizations per U2OS cell. Data from three independent experiments is shown as mean ± SEM from minimum 200 nuclei per experiment. Statistical evaluation was performed by one-way ANOVA test. ns: non-significant (*P* > .9999); ** *P *= .0032; .0035; .0062; .0092; .0052; **** *P* < .0001. (**D**) Model showing that TRF2–RAP1 inhibits telomere HDR by promoting BLM-mediated telomere R-loop removal. RAD51AP1 and TERRA-dependent R-loops promote RAD51-mediated telomere D-loop formation. The TRF2–RAP1 complex and TRF2–BLM interaction are required to promote BLM helicase-mediated unwinding of telomere R-loops and then D-loops. The RAP1–TRF2–BLM complex represses HDR on telomeres by removing R-loops to inhibit telomere D-loop formation.

In our previous study [15], we found that telomere-telomere clustering induces >8-fold increase in the brightness of telomere foci that we term UTs. We have previously shown that BLM-deficient U2OS cells expressing the TRF2^ΔB, L288R^ mutant displayed significantly increased number of UTs [19]. Since the TRF2^B^ cooperates with RAP1 to repress telomere R-loop formation on UTs [15], we next asked whether the RAP1–TRF2–BLM complex is required to repress telomere R-loop accumulation on UTs *in vivo*. U2OS cells expressing either shControl-vector or shBLM were reconstituted with either vector or shTRF2 plus TRF2^ΔB, L288R^ to generate UTs [15, 19]. The presence of R-loops on UTs was examined using the S9.6 antibody that recognized DNA–RNA hybrids [15, 51]. The merged PNA-FISH images with telomere (red) and R-loop (green) yield a yellow color, indicating UT/R-loop co-localization. We found that reconstitution with shTRF2 and TRF2^ΔB, L288R^ led to the co-localization of R-loops on UTs in ∼8.1% U2OS cells, with an average 0.33 UT/R-loop co-localization per cell. Depletion of BLM in cells expressing shTRF2 and TRF2^ΔB, L288R^ resulted in a significant increase in R-loop localization on UTs (Fig. 7A and C and Supplementary Fig. S3A), suggesting that BLM plays a crucial role in R-loop resolution on telomeres *in vivo*. RNase H treatment dramatically reduced R-loop co-localization with UTs (Supplementary Fig. S3B and C), confirming the specificity of S9.6 antibody for DNA–RNA hybrids. We also reconstituted WT BLM, BLM^3A^, or BLM^P690L^ mutants defective in TRF2 interaction, or the helicase-dead BLM^K695R^ mutant [19], in U2OS cells in which endogenous BLM was depleted by shBLM (Supplementary Fig. S3A). We found that reconstitution of WT BLM in BLM-deficient U2OS cells expressing shTRF2 and TRF2^ΔB, L288R^ reduced R-loop co-localization on UTs to levels observed in control cells expressing TRF2^ΔB, L288R^ (Fig. 7B and C). In contrast, reconstitution with either BLM^3A^, BLM^P690L^, or BLM^K695R^ mutants was unable to repress R-loop localization on UTs to levels observed in control cells (Fig. 7B and C and Supplementary Fig. S3A). These results support our biochemical data that the helicase activity of BLM and its interaction with TRF2–RAP1 are important to repress R-loop formation on telomeres *in vivo*. Our findings highlight a novel mechanism of the TRF2–RAP1–BLM complex to remove R-loops and inhibit D-loop formation on telomeres, thus repressing HDR-induced UT formation in U2OS cells (Fig. 7D).

## Discussion

Previous research revealed that RAD51AP1 binds to local RNA transcripts to drive the formation of R-loops, enhancing RAD51-mediated generation of D-loops to stimulate HDR [21]. RAD51AP1 promotes R-loop formation and enables RAD51 to form D-loops by facilitating the invasion of local RNA transcripts into donor dsDNA to form DR-loop intermediates that stimulate HDR [21]. At telomeres, RAD51 promotes telomere D-loop formation and physically interacts with TERRA to catalyze its association with telomeres by forming R-loops [25]. RAD51AP1 enables TERRA to form telomere R-loops more efficiently than RAD51 [22, 23]. We have previously shown that cells lacking TRF2^B^ and functional RAP1 display HDR-mediated telomere clustering, resulting in the formation of telomere D-loops and UTs [15, 19]. TRF2^B^ cooperates with RAP1 to repress telomere R-loop formation in UTs [15]. However, how TRF2–RAP1 represses TERRA R-loop-mediated telomere HDR is unclear.

In this study, we found that RAD51AP1 facilitates the invasion of TERRA transcripts into donor telomeric dsDNA, facilitating the generation of TERRA-R-loops. Telomere ssDNA and TERRA invade into telomere donor dsDNA to form DR-loop intermediates required for RAD51-mediated D-loop formation. We found that long TERRA transcripts generated significantly more D-loops compared to short TERRA transcripts. Mechanistically, RAD51-telomere ssDNA filaments preferentially captured telomere TERRA R-loops over telomere dsDNA to form D-loops. We also discovered that TRF2’s interaction with RAP1 increased the complex’s association with telomere R-loops. Both TRF2–RAP1 and TRF2–BLM interactions are essential to promote BLM-mediated unwinding of telomere R-loops. BLM preferentially released TERRA over telomere ssDNA from telomeric DR-loops, and the unwinding of telomere DR-loops by the RAP1–TRF2–BLM complex efficiently inhibited HDR at telomeres (Fig. 7D). Importantly, BLM-deficient U2OS cells and cells expressing BLM mutants defective in TRF2 binding accumulate telomere R-loops on UTs. Our results highlight a novel regulatory mechanism in which the TRF2–RAP1–BLM complex represses HDR on telomeres by removing TERRA R-loops to inhibit D-loop formation.

Accumulation of R-loops at telomeres promotes HDR and represents a source of telomere replication stress [20, 52]. TRF2 has been shown to bind to TERRA and telomere R-loops [53, 54] to stimulate telomeric DNA–RNA hybrid formation, while TRF1 is required to counteract TRF2-mediated TERRA R-loop formation [40, 54]. In addition, oxidative stress-induced DNA damage at telomeres triggers TRF2-dependent R-loop formation [55]. However, TRF2 has also been shown to inhibit R-loop formation by recruiting the nucleolar protein TCOF1 to telomeres to suppress TERRA transcription [56]. We have shown previously that TRF2 interacts with the RNA–DNA hybrid processing proteins ADAR1p110 and DDX2 to repress TERRA R-loop formation on UTs [15]. In our present study, we found that TRF2’s interaction with RAP1 is required for TRF2 to interact with TERRA R-loops. In addition, we found that BLM’s helicase activity and its binding to TRF2 are both essential to unwind TERRA R-loops. Our results suggest that shelterin components TRF2 and RAP1 cooperate with BLM to modulate telomere HDR by regulating D-loop formation. Specifically, the BLM–TRF2–RAP1 complex is critical to inhibit D-loop formation by removing TERRA R-loops from telomeres. This notion is further supported by our observation that cells expressing either the helicase-dead BLM mutant or BLM mutants unable to interact with TRF2 experience elevated R-loop formation at telomeres (Fig. 7A).

## Supplementary Material

gkag272_Supplemental_File

## Data Availability

All data are incorporated into the article and its online supplementary material.
