# Supplementary material for: TRF2–RAP1 inhibits homology-directed repair of telomeres by promoting BLM-mediated removal of telomere R-loops
Source: Nucleic Acids Res. 2026 Mar 31;54(6):gkag272. doi: 10.1093/nar/gkag272 (PMC13036488; doi:10.1093/nar/gkag272)
Supplement: gkag272_Supplemental_File [file gkag272_supplemental_file.pdf]

Supplementary data

TRF2-RAP1 inhibits homology-directed repair of telomeres by promoting BLM-mediated removal of telomere R-loops

Fengshan Liang and Sandy Chang

Suppl. Figure 1

A

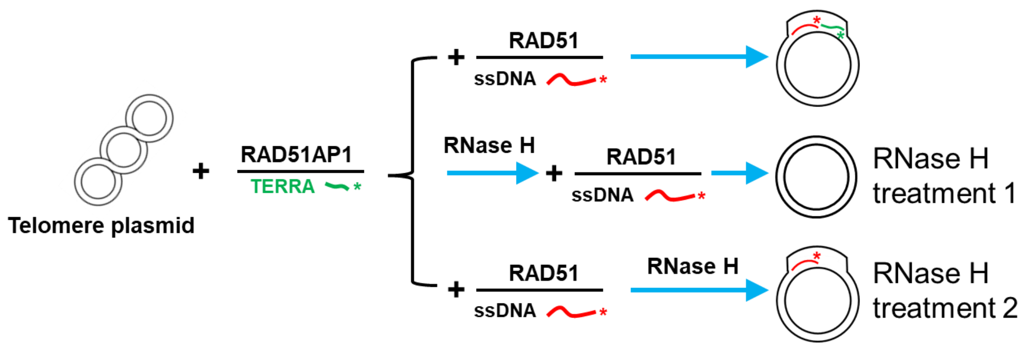

B

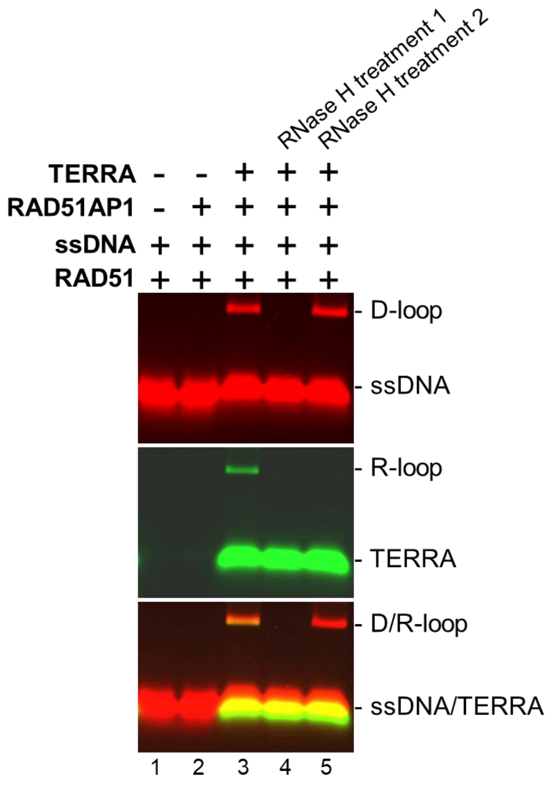

Suppl. Figure 2

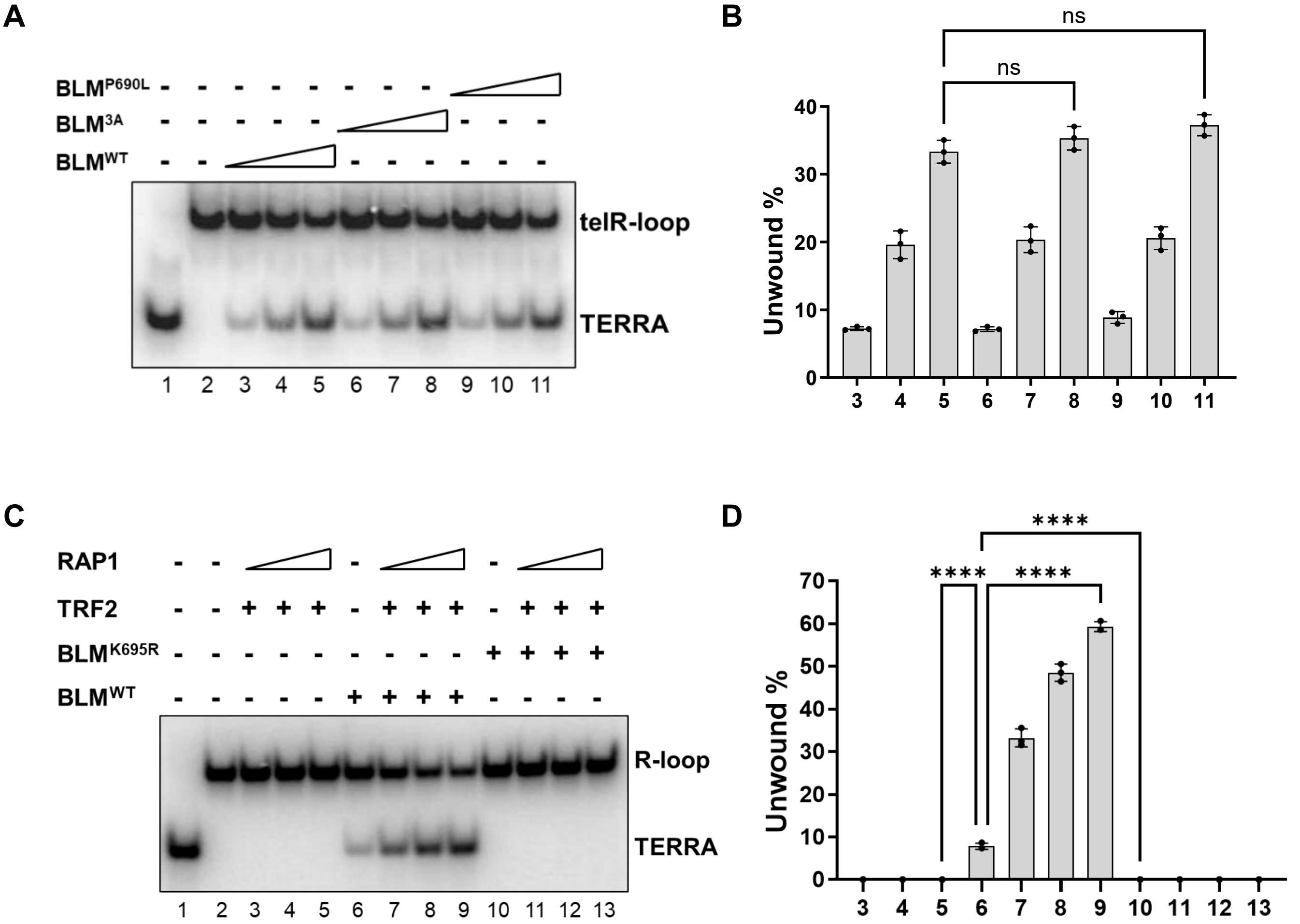

Suppl. Figure 3

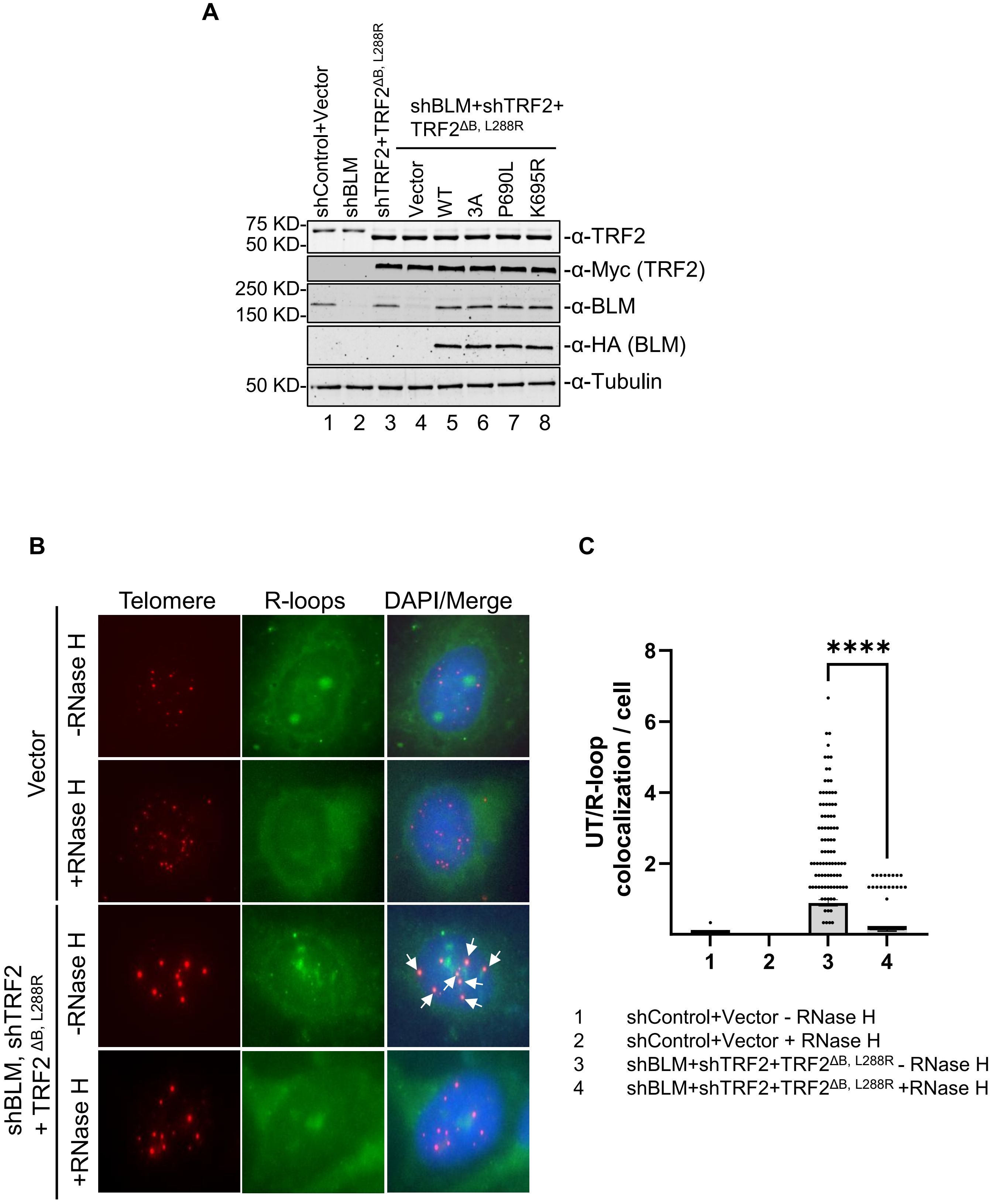

Supplementary figure legend

Supplementary Figure 1. RNase H treatment after D/R-loop formation impacts R-loops but not D-loops

A Schematic of the RAD51/ssDNA and RAD51AP1/TERRA-induced telomeric D/R-loop assay with and without RNase H treatment. IRDye-700-labeled telomeric G-rich ssDNA (red) was first incubated with purified RAD51 protein to form RAD51/ssDNA filaments. RAD51AP1 and IRDye-800-labeled TERRA (green) were incubated with the telomere plasmid to form R-loops. The samples were treated with RNase H before or after adding RAD51/ssDNA filaments. ssDNA or TERRA invasion into the plasmid formed telomeric D/R-loops. Red or green asterisks indicate the labeled ends of ssDNA or TERRA.

B RAD51AP1, TERRA and the indicated combinations were tested for their ability to enhance telomeric D-loop formation with RNase H treatment. In lane 4, the sample was treated with RNase H (0.5 unit) for 10 min before adding RAD51/ssDNA filaments. In lane 5, the sample was treated with RNase H after adding RAD51/ssDNA filaments. D/R-loop formation was visualized by 1% agarose gel electrophoresis.

Supplementary Figure 2. BLM^{3A} and BLM^{P690L} mutants possess intact telomeric R-loop unwinding activity

A WT BLM and BLM mutants (3A or P690L) all possess equal telomere R-loop unwinding activity. BLM proteins (20, 40, 80 nM) were incubated with telomere R-loops (2.5 nM) and BLM unwinding activity was examined. ^{32}P-labled TERRA and R-loops were loaded as molecular weight markers (lanes 1 and 2) and resolved in native polyacrylamide gel.

B Quantification of the percentages of unwound R-loops in (**A**) from three independent experiments is shown as mean ± S.D. Statistical difference was evaluated via ANOVA test. ns: nonsignificant (p=0.76; 0.08).

C The helicase activity of BLM is required for R-loop unwinding. The effect of TRF2-RAP1 on the ability of WT and the helicase-dead BLM^{K695R} mutant to unwind telomeric R-loops were tested. ^{32}P-labeled TERRA and R-loops were loaded as size markers (lanes 1 and 2) and resolved by native-PAGE.

D Quantification of the percentages of unwound R-loops as mean ±S.D. from three independent experiments. Statistical evaluation was performed by ANOVA test. **** P < 0.0001.

Supplementary Figure 3. The specificity of S9.6 antibody for DNA-RNA hybrids on UTs

A U2OS cells expressing TRF2$^{\Delta B,\ L288R}$ were infected with shControl, shBLM or shTRF2 and reconstituted with shBLM-resistant HA-tagged cDNAs encoding WT or mutant of BLM. Protein levels in cells were quantified by Western blotting.

B IF-FISH images showing the loss of R-loop signal (green) on UTs after 5U RNase H treatment for 2 hr on fixed cells. White arrow: co-localization of R-loops on UTs.

C Data from (**B**) was analyzed to determine the number of UT/R-loop co-localization per cell. The mean of three independent experiments ± SEM from a minimum 200 nuclei per experiment. Statistical evaluation was performed by one-way ANOVA test. **** P <0.0001.

Table S1 Oligonucleotides used in this study

Name	Sequence (5'-3')	Assay
Tel90	GGTTAGGGTTAGGGTTAGGGTTAGGGTTAGGGTTAGGGTTAGGGT TAGGGTTAGGGTTAGGGTTAGGGTTAGGGTTAGGGTTAGGGTTAG	Telomere D-loop; RAD51 filament stabilization assay
TERRA	UUAGGGUUAGGGUUAGGGUUAGGGUUAGGG	Telomere D/R-loop assay; R-loop generation
TDR1	AGCTCCTAGGGTTACAAGCTTCACTAGTTAGGGTTAGGGTTAGGGT TAGGGTTAGGGCCTACACATGTAGGGTTGATCAGC	Telomere dsDNA generation
TDR2	GCTGATCAACCCTACATGTGTAGGCCCTAACCCTAACCCTAACCCT AACCCTAACTAGTGAAGCTTGTAACCCTAGGAGCT	Telomere dsDNA and R-loop generation
TDR3	AGCTCCTAGGGTTACAAGCTTCACTAGCAGAGCGCGTCACAGTCA GAGTCACAGCAGCCTACACATGTAGGGTTGATCAGC	Telomere R-loop generation
